# Supplementary material for: Structural basis for the specificity of PPM1H phosphatase for Rab GTPases
Source: EMBO Rep. 2021 Sep 28;22(11):e52675. doi: 10.15252/embr.202152675 (PMC8567228; doi:10.15252/embr.202152675)
Supplement: Supplementary file 1 — Expanded View Figures PDF [file EMBR-22-e52675-s002.pdf]

Expanded View Figures

Figure EV1. The "loop deletion" variant of PPM1H is active in vitro and in cells.

A HEK293 cells overexpressing indicated constructs were treated ± 200 nM MLi-2 for 90 min and then lysed. 10 μg whole cell lysate was subjected to immunoblot analysis with the indicated antibodies at 1 μg/ml final concentration, and membranes were analysed using the OdysseyClx Western blot imaging system. Each lane represents cell extract obtained from a different dish of cells (two replicates per condition without MLi-2 treatment, one replicate per condition with MLi-2 treatment). The "loopDEL" variant is the segment 33–514 with the region 188-226 replaced by the sequence "GSGS".

B *In vitro* assay of PPM1H activity using a PhosTag gel. Substrate pRab8a (GTP form, 10 μg) was incubated with WT PPM1H ± loopDEL for 15 min at room temperature. The full-length variant was fused to maltose-binding protein (MBP). The "loopDEL" variant was from 33 to 514 and was used for crystallization studies. A conventional 12% SDS–PAGE is shown in parallel lanes below the PhosTag gel.

C Structure-based sequence alignment of human PPMs from their associated PDB files using Chimera software (Pettersen *et al*, 2004). The PDB codes are 4ra2 (PPM1A; (Pan *et al*, 2015)), 2p8e (PPM1B; (Almo *et al*, 2007)) and 2iq1 (PPM1K; (Almo *et al*, 2007)). Residue His31 of PPM1H is in brackets since the first two residues (His-Met) arise from a cloning artefact. Secondary structures of PPM1H and PPM1A are above and below the sequences, respectively. Colours of secondary structures correspond to the scheme in Fig 1. The black triangle is a loop in PPM1H (188-226) that has been removed to simplify the alignment. The C-terminal α-helical domain of PPM1A is blue. Red circles are aspartate residues that directly coordinate metal ions.

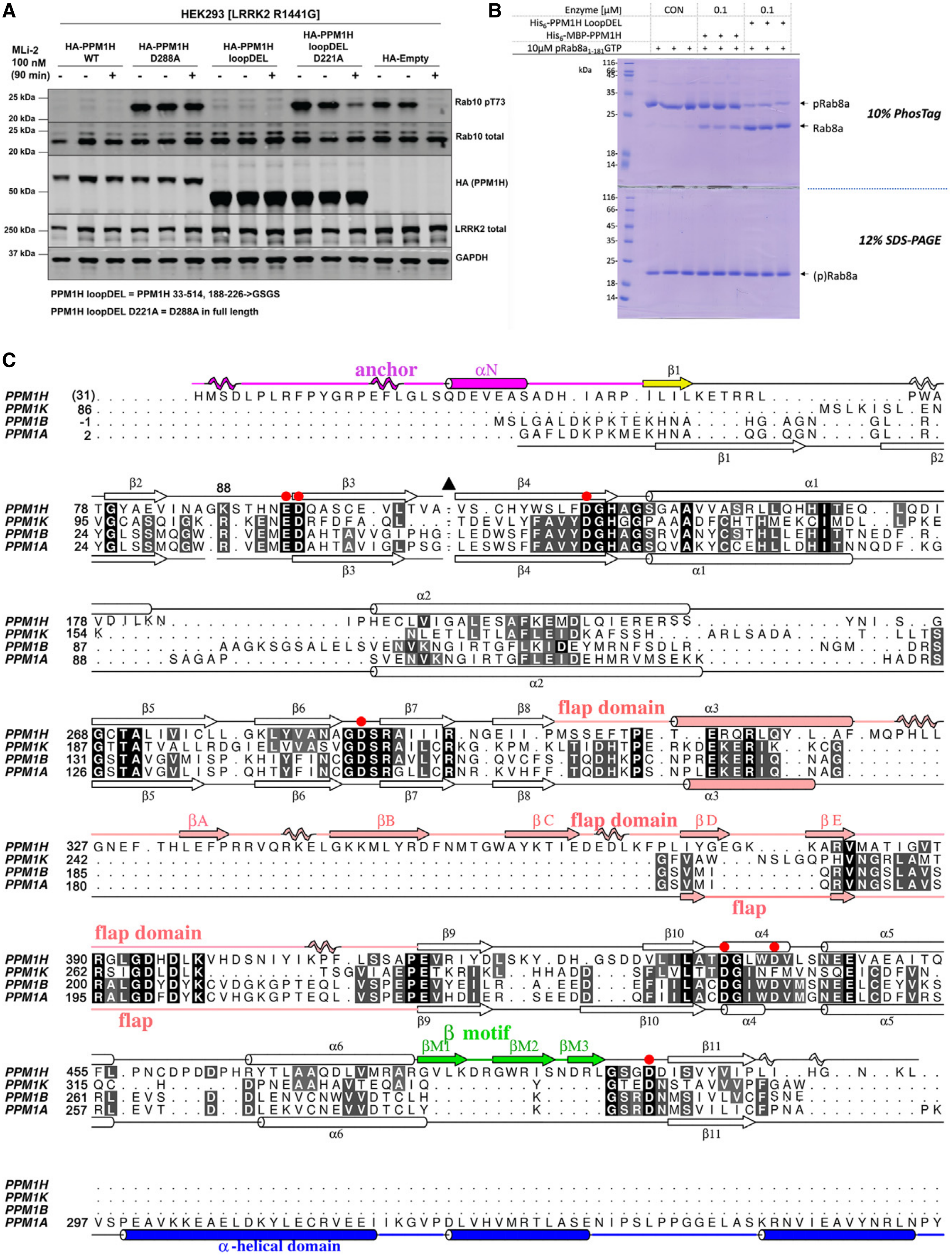

Figure EV1.

 © 2021 The Authors

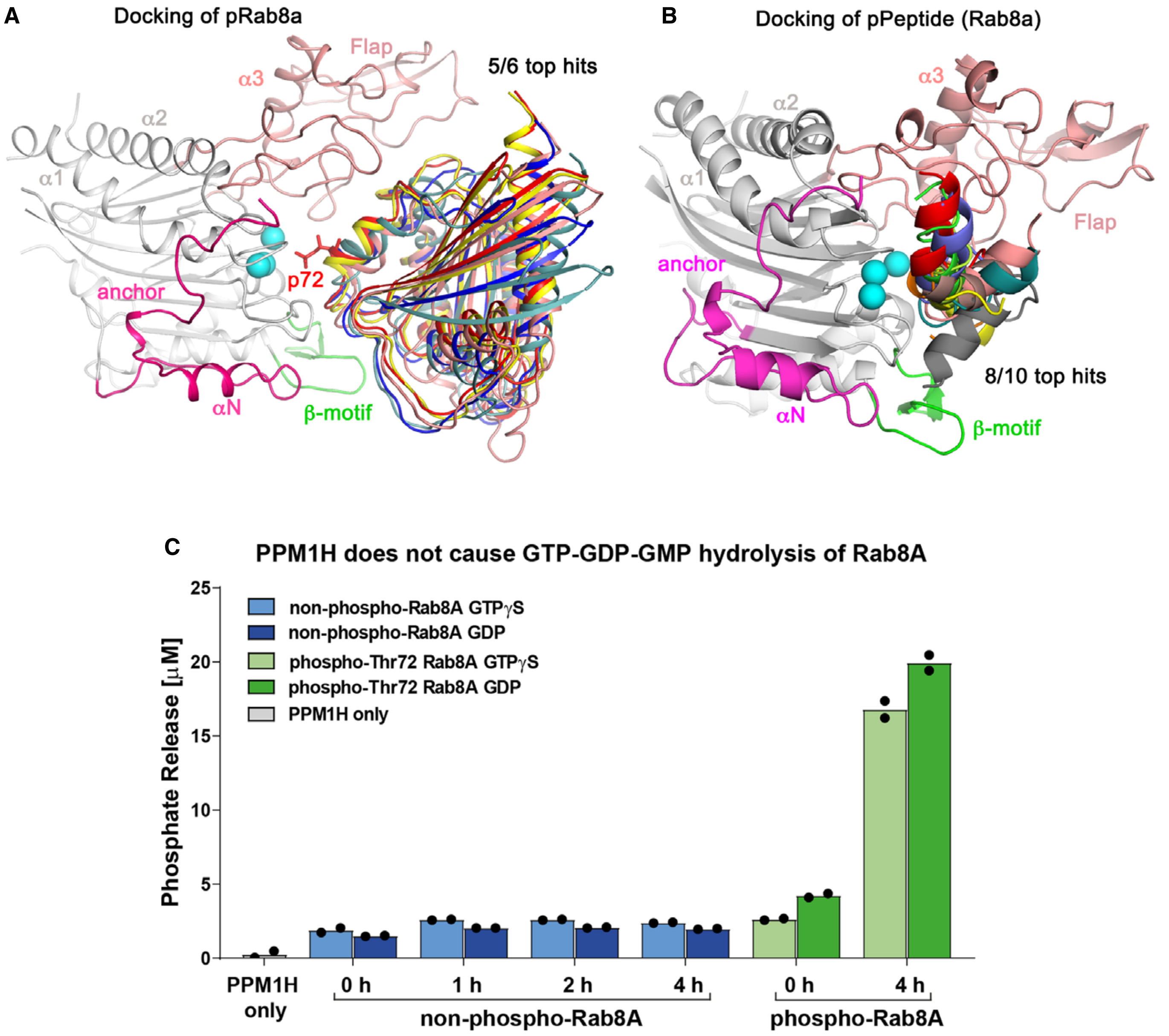

Figure EV2. Docking of PPM1H with phosphorylated Rab8a.

A Docking of 5/6 top solutions for MnPPM1H^{WT}-LD complex with pRab8a using Haddock software. A stick model for pThr72 (p72, red) is shown for one of the hits, adjacent to the Mn^{2+} ions. All of these docking poses pack against the flap domain.

B Docking of the switch 2 phosphopeptide against MnPPM1H^{WT}-LD. The solutions are in addition to those shown in Fig 2 and are shown for completeness of the docking results. However, these 8 poses are sterically incompatible with the active site in the context of the full-length pRab8a protein.

C 50 nM recombinant wild-type PPM1H was incubated with 32 μM of non-phospho- and pThr72-phosphorylated Rab8a (GTPγS or GDP) for 1, 2 or 4 h. Phosphate release was measured as described in Materials and Methods. The experiments were repeated 2 times.

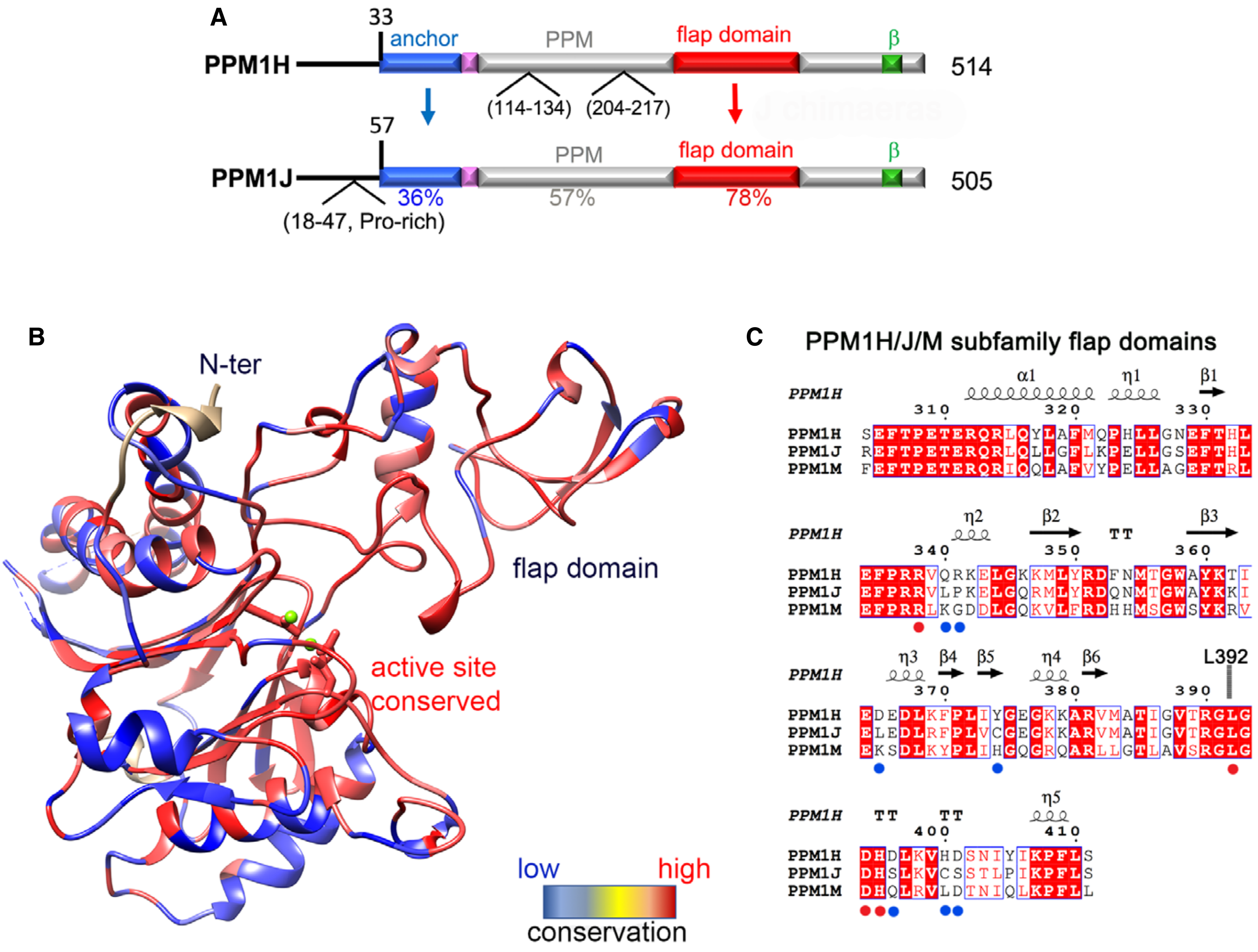

Figure EV3. Design of PPM1H/J chimaeras to probe substrate specificity.

A Domain organization of PPM1H and PPM1J with locations of predicted flexible loops. Sequence identities within domains are indicated below the alignment.

B Heat map of PPM1H and PPM1J sequence diversity superimposed onto the ribbon model of PPM1H. The rectangular bar displays the variation from low (blue) to high (red) sequence identities.

C Sequence alignment of the flap domains of PPM1H and PPM1J. Secondary structure annotations of PPM1H are above the alignment. Blue and red circles below the alignment indicate conserved and non-conserved residues subjected to site-directed mutagenesis.

 © 2021 The Authors
