# Supplementary material for: Structural basis for the specificity of PPM1H phosphatase for Rab GTPases
Source: EMBO Rep. 2021 Sep 28;22(11):e52675. doi: 10.15252/embr.202152675 (PMC8567228; doi:10.15252/embr.202152675)
Supplement: Supplementary file 2 [file EMBR-22-e52675-s001.pdf]

EMBO reports

Structural basis for the specificity of PPM1H phosphatase for Rab GTPases

Dieter Waschbusch, Kerryn Berndsen, Pawel Lis, Axel Knebel, Yuko Lam, Dario Alessi, and Amir Khan
DOI: 10.15252/embr.202152675

Corresponding author(s): Amir Khan (amirrafk@tcd.ie) , Dario Alessi (d.r.alessi@dundee.ac.uk)

Review Timeline:

Submission Date:	15th Feb 21
Editorial Decision:	9th Apr 21
Revision Received:	2nd Jul 21
Editorial Decision:	10th Aug 21
Revision Received:	16th Aug 21
Accepted:	27th Aug 21

Editor: Martina Rembold

Transaction Report:

(Note: With the exception of the correction of typographical or spelling errors that could be a source of ambiguity, letters and reports are not edited. Depending on transfer agreements, referee reports obtained elsewhere may or may not be included in this compilation. Referee reports are anonymous unless the Referee chooses to sign their reports.)

1st Editorial Decision 9th Apr 2021

Dear Dr. Khan

Thank you for the submission of your research manuscript to our journal. We have now received the full set of referee reports that is copied below.

As you will see, while referee 2 and 3 are rather positive and support a revision for EMBO reports, referee 1 remains critical regarding the conclusiveness of the current dataset. It will be essential to strengthen your conclusions and to address all referee concerns, i.e., to provide phosphatase data for Rab10, a PPM1H chimeria with the PPM1J flap domain and more insight into the relevant residues. While I agree that a co-crystal with substrate would strengthen the work substantially, I also value the suggestion from referee 3 to make a docking model of PPM1H with the phosphorylated peptide, should co-crystallization be difficult to achieve.

Based on this evaluation, we would like to invite you to revise your manuscript with the understanding that the referee concerns (as detailed above and in their reports) must be fully addressed and their suggestions taken on board. Please address all referee concerns in a complete point-by-point response. Acceptance of the manuscript will depend on a positive outcome of a second round of review. It is EMBO reports policy to allow a single round of revision only and acceptance or rejection of the manuscript will therefore depend on the completeness of your responses included in the next, final version of the manuscript.

We invite you to submit your manuscript within three months of a request for revision. This would be July 9th in your case. However, we are aware of the fact that many laboratories are not fully functional due to COVID-19 related shutdowns and we have therefore extended the revision time for all research manuscripts under our scooping protection to allow for the extra time required to address essential experimental issues. Please contact us to discuss the time needed and the revisions further.

***IMPORTANT NOTE: we perform an initial quality control of all revised manuscripts before re-review. Your manuscript will FAIL this control and the handling will be DELAYED if the following APPLIES:

1) A data availability section is missing.
2) Your manuscript contains error bars based on n=2. Please use scatter blots showing the individual datapoints in these cases. The use of statistical tests needs to be justified.

When submitting your revised manuscript, please carefully review the instructions that follow below. Failure to include requested items will delay the evaluation of your revision.***

When submitting your revised manuscript, we will require:

1) a .docx formatted version of the manuscript text (including legends for main figures, EV figures and tables). Please make sure that the changes are highlighted to be clearly visible.

2) individual production quality figure files as .eps, .tif, .jpg (one file per figure).
Please download our Figure Preparation Guidelines (figure preparation pdf) from our Author

Guidelines pages
https://www.embopress.org/page/journal/14693178/authorguide for more info on how to prepare your figures.

3) a .docx formatted letter INCLUDING the reviewers' reports and your detailed point-by-point responses to their comments. As part of the EMBO Press transparent editorial process, the point-by-point response is part of the Review Process File (RPF), which will be published alongside your paper.

4) a complete author checklist, which you can download from our author guidelines (). Please insert information in the checklist that is also reflected in the manuscript. The completed author checklist will also be part of the RPF.

5) Please note that all corresponding authors are required to supply an ORCID ID for their name upon submission of a revised manuscript (). Please find instructions on how to link your ORCID ID to your account in our manuscript tracking system in our Author guidelines
()

6) We replaced Supplementary Information with Expanded View (EV) Figures and Tables that are collapsible/expandable online. A maximum of 5 EV Figures can be typeset. EV Figures should be cited as 'Figure EV1, Figure EV2" etc... in the text and their respective legends should be included in the main text after the legends of regular figures.

- For the figures that you do NOT wish to display as Expanded View figures, they should be bundled together with their legends in a single PDF file called *Appendix*, which should start with a short Table of Content. Appendix figures should be referred to in the main text as: "Appendix Figure S1, Appendix Figure S2" etc. See detailed instructions regarding expanded view here:

- Additional Tables/Datasets should be labeled and referred to as Table EV1, Dataset EV1, etc. Legends have to be provided in a separate tab in case of .xls files. Alternatively, the legend can be supplied as a separate text file (README) and zipped together with the Table/Dataset file.

7) Data availability (structures): The accession numbers and database should be listed in a formal "Data Availability " section (placed after Materials & Method) that follows the model below (see also < https://www.embopress.org/page/journal/14693178/authorguide#dataavailability>). Please note that the Data Availability Section is restricted to new primary data that are part of this study and please remember to provide a reviewer password if the datasets are not yet public.

Data availability

The datasets (and computer code) produced in this study are available in the following databases:

- RNA-Seq data: Gene Expression Omnibus GSE46843
(https://www.ncbi.nlm.nih.gov/geo/query/acc.cgi?acc=GSE46843)
- [data type]: [name of the resource] [accession number/identifier/doi] ([URL or identifiers.org/DATABASE:ACCESSION])

*** Note - All links should resolve to a page where the data can be accessed. ***

8) We would also encourage you to include the source data for figure panels that show essential data. Numerical data should be provided as individual .xls or .csv files (including a tab describing the data). For blots or microscopy, uncropped images should be submitted (using a zip archive if multiple images need to be supplied for one panel). Additional information on source data and instruction on how to label the files are available .

9) Our journal encourages inclusion of *data citations in the reference list* to directly cite datasets that were re-used and obtained from public databases. Data citations in the article text are distinct from normal bibliographical citations and should directly link to the database records from which the data can be accessed. In the main text, data citations are formatted as follows: "Data ref: Smith et al, 2001" or "Data ref: NCBI Sequence Read Archive PRJNA342805, 2017". In the Reference list, data citations must be labeled with "[DATASET]". A data reference must provide the database name, accession number/identifiers and a resolvable link to the landing page from which the data can be accessed at the end of the reference. Further instructions are available at .

10) Regarding data quantification
The following points must be specified in each figure legend:
- the name of the statistical test used to generate error bars and P values,
- the number (n) of independent experiments (please specify technical or biological replicates) underlying each data point,
- the nature of the bars and error bars (s.d., s.e.m.)
Discussion of statistical methodology can be reported in the materials and methods section, but figure legends should contain a basic description of n, P and the test applied.
- Please also include scale bars in all microscopy images.

11) As part of the EMBO publication's Transparent Editorial Process, EMBO reports publishes online a Review Process File to accompany accepted manuscripts. This File will be published in conjunction with your paper and will include the referee reports, your point-by-point response and all pertinent correspondence relating to the manuscript.

You are able to opt out of this by letting the editorial office know (emboreports@embo.org). If you do opt out, the Review Process File link will point to the following statement: "No Review Process File is available with this article, as the authors have chosen not to make the review process public in this case."

We would also welcome the submission of cover suggestions, or motifs to be used by our Graphics Illustrator in designing a cover.

I look forward to seeing a revised version of your manuscript when it is ready. Please let me know if you have questions or comments regarding the revision.

Yours sincerely

Martina Rembold, PhD
Senior Editor
EMBO reports

****

Referee #1:

The manuscript from Waschbüsch et al. describes the mechanism for substrate recognition of the PPM phosphatase, PPM1H. The authors showed crystal structure of PPM1H and analyzed the role of a specific flap domain located in the conserved catalytic domain on its substrate recognition for Rab8a. They suggested that the residue R338 in the domain is involved in the interaction with phospho-Rab8a protein by using a docking model. They also showed that a PPM1J chimera protein with the PPM1H flap domain dephosphorylated pThr72 of Rab8a. Based on these findings, the authors claim that the flap domain of PPM1H has a specific interaction with the pRab8a protein. This work has some novelty in structural analysis. However, there are significant flaws in the lack of data and interpretations of results that preclude publication in EMBO Reports.

Specific comments are indicated below:

Major points:
1. The sequences of the flap domains in PPM1J and PPM1H are highly homologous. Therefore the authors should provide experimental data showing which particular residue(s) in the flap domain of PPM1H are critical for specific Rab recognition.
2. The authors used Rab10 for cell assay and Rab8a for docking and phosphatase assays. Although the sequences of Rab10 and Rab8a fragments used in this study are very similar, there is difference including the vicinity of the dephosphorylation site. Due to high homology of the flap sequences in PPM1J and PPM1H, the authors should show results of the phosphatase assay using phosphorylated Rab10 and Rab10 phosphopeptides.
Otherwise the authors should show the co-crystal structure of PPM1H and the substrate.
4. The authors should show a PPM1H chimera with the PPM1J flap domain did not dephosphorylates pRab8a protein.
5. The authors concluded that R338 residue in the flap domain of PPM1H is associated with Rab8a binding. PPM1J WT did not dephosphorylate Rab although R338 residue is conserved in the flap domain of PPM1J. The authors should discuss the results of R338 mutant and of the PPM1J chimera protein.

Referee #2:

The study of Dieter Waschbüsch and colleagues show interesting new data on the mechanism how PPM1H counteracts the LRRK2 mediated phosphorylation on Rab proteins. The study describes a crystal structure of PPM1H. Additionally to a conserved catalytic domain, they could identify a unique 110-residue FLAP domain, which distantly resembles Tudor domains that interact with histones. All other members of the human PPM family with solved structures have shorter Flap domains. The work demonstrates that the Flap domain is responsible for substrate specificity towards phosphorylated Rabs by several approaches, i.e. substrate docking, a mutational analysis as well as the generation of a chimeric protein consisting of the PPM1J enzymatic core and the PPM1H Flap domain. Furthermore, the authors determined the kinetical parameters for the PPM1H catalyzed hydrolysis reaction.
The work is technically sound and provides interesting new details about the hydrolysis mechanism and the interaction of PPMH1 with its substrate phospho-T72 Rab8a. I can therefore recommend

the work for publication with minor revisions.

Detailed critique:

1.) There is a mismatch between the PPM1H mutants mentioned in the text (page 7, line 20 following) and those shown in figure 2C: K88A and R338A are not shown in figure 2c but in the supplemental figure EV2B, instead.

2.) The authors have questioned in which nucleotide-state phospho-Rab8a is dephosphorylated by PPM1H (Figure 2D, 3. Panel). For this purpose, they assayed GDP-bound vs. GTPgS-bound phosphor-Rab8a determining the free phosphate by the Malachitgreen assay. They could demonstrate that both forms are dephosphorylated by PPM1H, however with a different kinetics. The GDP-bound form had a higher Km value and a 2-fold higher vmax compared to GTPgS-pRab8a. Given that, only the free phosphate was determined, can the authors rule out that the GDP was hydrolyzed by the phosphatase? The latter could contribute to the free phosphate levels potentially leading to a misinterpretation of the kinetical parameters determined for the GDP-bound Rab8a. Unphosphorylated GDP-bound RAB8a could be used as a potential control.

Referee #3:

Summary

1. Does this manuscript report a single key finding? YES

The manuscript reports the first structural information on the Rab-specific phosphatase PPM1H and pinpoints the basis of substrate specificity within its flap domain.

2. Is the reported work of significance (YES), or does it describe a confirmatory finding or one that has already been documented using other methods or in other organisms etc (NO)? YES

3. Is it of general interest to the molecular biology community? YES

Mutations in LRRK2 are associated with Parkinson's disease and increase its kinase activity toward Rab substrates. The protein phosphatase PPM1H counteracts this effect and insight in its structure and specificity is hence of importance to understand the mechanisms underlying LRRK2-associated disease.

4. Is the single major finding robustly documented using independent lines of experimental evidence (YES), or is it really just a preliminary report requiring significant further data to become convincing, and thus more suited to a longer¬ format article (NO)? YES

Report

Mutations in LRRK2 are among the most common causes of inherited Parkinson's disease. LRRK2 possesses Ser/Thr protein kinase activity and several Parkinson mutations lead to an increase in kinase activity. In the last few years several Rab GTPases (including Rab8a and Rab10) were identified as the physiological substrates of LRRK2, where LRRK2 specifically phosphorylates T72 within their Switch 2 region. Rab phosphorylation on T72 can be counteracted by the Ser/Thr

phosphatase PPM1H. Although, structures of other members of this phosphatase family have previously been solved, no structure of PPM1H was available up to this point and the mechanism underlying the specificity of PPM1H for phosphorylated Rabs, in contrast to other close related phosphatases, remained elusive. In this manuscript, Waschbüsch and colleagues present the first crystal structures of PPM1H up to about 2.5 Å resolution, showing that in comparison to related enzymes PPM1H possesses a more extended so-called "flap domain" inserted within the catalytic domain. In a very elegant approach, they construct chimeric enzymes by mix-and-matching N-terminal, catalytic and flap domains between PPM1H and the related PPM1J. The latter shows only very basal activity toward the Rab proteins. With this experiment the authors can unequivocally pinpoint the specificity-determining region within the flap domain (providing one unclarity remaining, as described under my remarks). Overall, this is a focused and elegant study, which is technically sound and which is also clearly described and represented.

A few comments and questions remain for the authors to address:

- The authors describe that the Rab-specificity of PPM1H resides within its flap domain, since a chimaera consisting of the catalytic domain of PPM1J and the flap domain of PPM1H is fully active toward Rab8a. This is also in agreement with the PPM1H-Rab docking model, showing that the flap domain folds over the switch regions and GDP/GTP-binding pocket of Rab. However, intriguingly, the authors show that the flap region also determines specificity for a phosphorylated peptide spanning the region around T72. From the docking model is it less clear how this can be explained. Can the authors attempt to make a docking model of PPM1H with this peptide? This would be very useful to include in the paper. Of course an experimental crystal structure of the PPM1H-peptide complex would even be better, but this reviewer fully understands that the latter most probably has been already attempted without success and will therefore probably not be possible within a reasonable timeframe.

As a sidenote, it is quite striking that the nucleotide state of Rab8A has a relatively small impact on the PPM1H catalytic parameters (Fig. 2D), considering the position of the flap regions in comparison to the Rab switch regions .

- For a comparison of the initial rates of WT and mutant PPM1H toward Rab8a and peptide substrates, 16 µM Rab and 32 µM peptide is used. While for the latter this concentration is very close to the KM value, this is unclear for the peptide as kcat and KM values for the peptide are not reported. For a straightforward comparison of the effect of mutants (e.g. R338A) on Rab and peptide dephosphorylation, substrate concentrations should be used that relate in a similar way to their KM values, e.g. either below or above KM to assess the effect on kcat/KM or kcat, respectively.

Minor comments:
- The rationale for making the D288A mutant to increase the crystallisability of the protein is unclear to me (of course, as long as it works it is fine).

- Figure 2D right panel: the individual datapoints (initial rates at different substrate concentrations) should also be shown, rather than only showing the fitted curve.

Typo's:
- page 7, line 9: "phsopho" should be "phospho"
- page 21 (figure 2 legend): "WT and PPM1H (25 nM) were incubated" should probably be "WT and mutant PPM1H (25 nM) were incubated"
- p22, line 8: "Kcat" should be "kcat".

To obtain further evidence that the Flap domain of PPM1H functions as a substrate recognition domain for Rab GTPases, we have performed mass spectrometry crosslinking analyses of a substrate trapping PPM1H variant (D288A) complexed to phosphorylated Rab8a. The new data are included in Figure 2A and 2B. It demonstrates the mass spectrometry crosslinker DSBU (disuccinimidyl dibutyric urea) induces many crosslinks between the Flap domain of PPM1H and pRab8A, consistent with the Flap domain operating as a Rab8A recognition domain. The crosslinking data are also consistent with the top solutions from in silico docking calculations for the PPM1H-pRab8A complex shown in Figure 2C.

We have also included new multi-angle light scattering and mass photometry data demonstrating that PPM1H is a dimer in solution (Figure 3A, 3B, 3C). We exploited the structural data to develop a monomeric PPM1H variant by mutation of residues at the dimeric interface and demonstrate that this mutation moderately enhances PPM1H activity in vitro towards pRab8A without impacting activity towards peptide substrates (Fig 3D).

Further experiments and revisions we have undertaken to address the Reviewers constructive comments are described below.

Referee #1:

The manuscript from Waschbüsch et al. describes the mechanism for substrate recognition of the PPM phosphatase, PPM1H. The authors showed crystal structure of PPM1H and analyzed the role of a specific flap domain located in the conserved catalytic domain on its substrate recognition for Rab8a. They suggested that the residue R338 in the domain is involved in the interaction with phospho-Rab8a protein by using a docking model. They also showed that a PPM1J chimera protein with the PPM1H flap domain dephosphorylated pThr72 of Rab8a. Based on these findings, the authors claim that the flap domain of PPM1H has a specific interaction with the pRab8a protein.
This work has some novelty in structural analysis. However, there are significant flaws in the lack of data and interpretations of results that preclude publication in EMBO Reports.

Specific comments are indicated below:

Major points:
1. The sequences of the flap domains in PPM1J and PPM1H are highly homologous. Therefore the authors should provide experimental data showing which particular residue(s) in the flap domain of PPM1H are critical for specific Rab recognition.

To address the Reviewer’s critique, we have performed mutagenesis of three epitopes on the flap domain of PPM1H (Y374C, H400C+D401S, Q340L+R341P+D365L). The three sites are divergent among PPM1H, PPM1J and PPM1M (Fig EV4C). In addition, these 3 sites are predicted to be close to phospho-Rab8 in the docked model of the complex. We find that mutagenesis does not affect catalysis in vitro or in cells (Fig 5E/F). This may

suggest that the molecular basis for specificity is more complex than one or a few dominant epitopes. Also, residues that are conserved in H/J (e.g, R338 of PPM1H; Fig 5B/C) may nevertheless contribute to Rab recognition in the context of PPM1H. Specificity may involve conformational differences in the flap domain that are difficult to investigate through mutagenesis experiments. We have discussed these issues in the revised manuscript ('Mutagenesis' section of results).

2. The authors used Rab10 for cell assay and Rab8a for docking and phosphatase assays. Although the sequences of Rab10 and Rab8a fragments used in this study are very similar, there is difference including the vicinity of the dephosphorylation site. Due to high homology of the flap sequences in PPM1J and PPM1H, the authors should show results of the phosphatase assay using phosphorylated Rab10 and Rab10 phosphopeptides.
Otherwise the authors should show the co-crystal structure of PPM1H and the substrate.

We appreciate the reviewer raising the issue of different substrates in our initial submission. Rab10 is poorly expressed and difficult to purify in milligram amounts in its phosphorylated state, hence the focus on phospho-Rab8a for in vitro kinetic assays. For the modelling, we previously determined the structure of phospho-Rab8a in complex with an effector, hence the use of the protein for docking analyses with PPM1H. During revisions, we have overcome some of the technical issues in expression and phosphorylation of Rab10. This has enabled us to generate sufficient phospho-Rab10 for a qualitative assay of PPM1H hydrolysis (Fig 1D). This demonstrates that PPM1H efficiently dephosphorylates pRab10 under conditions where PPM1J displays no activity. We are still unable to generate milligram amounts of pRab10 due to insufficient expression and lack of stability, relative to pRab8A. We have compensated for this limitation by using phosphopeptides from both Rab8a and Rab10 in all kinetics experiments (Figs 2E, 3D, 4D, 5B). Our data demonstrate that PPM1H efficiently dephosphorylates the pRab8A and pRab10 peptides. Interestingly PPM1J appears to dephosphorylate pRab10 peptide more efficiently than pRab8A peptide albeit at a much lower rate than PPM1H.

Regarding the PPM1H-substrate complex, we are obviously keen on determining the structure. This is a significant challenge given the low affinity of complexes in vitro. We are engineering Rab proteins and peptides to enhance affinities, and also using both CryoEM and crystallography approaches to accelerate the process. However this project requires considerable effort and it is not achievable for the time frame of this study.

4. The authors should show a PPM1H chimera with the PPM1J flap domain did not dephosphorylates pRab8a protein.

We have performed this important experiment both in vitro and in cell assays. A PPM1H variant with the PPM1J flap domain ('H/J flap') is inactive against both phosphorylated Rab8a protein and peptide (Fig 4C-4D, emphasized with an orange colour). Cellular assays also show a significant loss of activity with this mutant (Fig 4E-4F).

5. The authors concluded that R338 residue in the flap domain of PPM1H is associated with Rab8a binding. PPM1J WT did not dephosphorylate Rab although R338 residue is conserved in the flap domain of PPM1J. The authors should discuss the results of R338 mutant and of the PPM1J chimera protein.

As mentioned in the response to point 1, we discuss that conserved residues may nevertheless contribute to pRab8a recognition in the context of the PPM1H flap domain. There are numerous examples of molecular recognition that involves distinct conformations of apparently conserved regions. One is the mechanism by which Rab GTPases recognize distinct effectors through highly conserved switch 1 and 2 regions.

Referee #2:

The study of Dieter Waschbüsch and colleagues show interesting new data on the mechanism how PPM1H counteracts the LRRK2 mediated phosphorylation on Rab proteins. The study describes a crystal structure of PPM1H. Additionally to a conserved catalytic domain, they could identify a unique 110-residue FLAP domain, which distantly resembles Tudor domains that interact with histones. All other members of the human PPM family with solved structures have shorter Flap domains. The work demonstrates that the Flap domain is responsible for substrate specificity towards phosphorylated Rabs by several approaches, i.e. substrate docking, a mutational analysis as well as the generation of a chimeric protein consisting of the PPM1J enzymatic core and the PPM1H Flap domain. Furthermore, the authors determined the kinetical parameters for the PPM1H catalyzed hydrolysis reaction.
The work is technically sound and provides interesting new details about the hydrolysis mechanism and the interaction of PPMH1 with its substrate phospho-T72 Rab8a. I can therefore recommend the work for publication with minor revisions.

Detailed critique:

1.) There is a mismatch between the PPM1H mutants mentioned in the text (page 7, line 20 following) and those shown in figure 2C: K88A and R338A are not shown in figure 2c but in the supplemental figure EV2B, instead.

In the revisions, K88A and R338A have been moved to Fig 5. We appreciate the reviewer pointing this out, the text now matches the figures.

2.) The authors have questioned in which nucleotide-state phospho-Rab8a is dephosphorylated by PPM1H (Figure 2D, 3. Panel). For this purpose, they assayed GDP-bound vs. GTPgS-bound phosphor-Rab8a determining the free phosphate by the Malachitgreen assay. They could demonstrate that both forms are dephosphorylated by PPM1H, however with a different kinetics. The GDP-bound form had a higher Km value and a 2-fold higher vmax compared to GTPgS-pRab8a. Given that, only the free phosphate was determined, can the authors rule out that the GDP was hydrolyzed by the phosphatase? The latter could contribute to the free phosphate levels potentially leading

to a misinterpretation of the kinetical parameters determined for the GDP-bound Rab8a. Unphosphorylated GDP-bound RAB8a could be used as a potential control.

A control experiment has been performed to show any background generation of free phosphate when PPM1H is incubated with guanosine nucleotides used in our assays (Fig EV2G). There is no significant hydrolysis of GTPγS and GDP when they are bound to non-phosphorylated Rab8a.

Referee #3:

Summary
1. Does this manuscript report a single key finding? YES

The manuscript reports the first structural information on the Rab-specific phosphatase PPM1H and pinpoints the basis of substrate specificity within its flap domain.

2. Is the reported work of significance (YES), or does it describe a confirmatory finding or one that has already been documented using other methods or in other organisms etc (NO)? YES

3. Is it of general interest to the molecular biology community? YES

Mutations in LRRK2 are associated with Parkinson's disease and increase its kinase activity toward Rab substrates. The protein phosphatase PPM1H counteracts this effect and insight in its structure and specificity is hence of importance to understand the mechanisms underlying LRRK2-associated disease.

4. Is the single major finding robustly documented using independent lines of experimental evidence (YES), or is it really just a preliminary report requiring significant further data to become convincing, and thus more suited to a longer¬ format article (NO)? YES

Report

Mutations in LRRK2 are among the most common causes of inherited Parkinson's disease. LRRK2 possesses Ser/Thr protein kinase activity and several Parkinson mutations lead to an increase in kinase activity. In the last few years several Rab GTPases (including Rab8a and Rab10) were identified as the physiological substrates of LRRK2, where LRRK2 specifically phosphorylates T72 within their Switch 2 region. Rab phosphorylation on T72 can be counteracted by the Ser/Thr phosphatase PPM1H. Although, structures of other members of this phosphatase family have previously been solved, no structure of PPM1H was available up to this point and the mechanism underlying the specificity of PPM1H for phosphorylated Rabs, in contrast to other close related phosphatases, remained elusive. In this manuscript, Waschbüsch and colleagues present the first crystal structures of PPM1H up to about 2.5 Å resolution, showing that in

comparison to related enzymes PPM1H possesses a more extended so-called "flap domain" inserted within the catalytic domain. In a very elegant approach, they construct chimeric enzymes by mix-and-matching N-terminal, catalytic and flap domains between PPM1H and the related PPM1J. The latter shows only very basal activity toward the Rab proteins. With this experiment the authors can unequivocally pinpoint the specificity-determining region within the flap domain (providing one unclarity remaining, as described under my remarks). Overall, this is a focused and elegant study, which is technically sound and which is also clearly described and represented.

A few comments and questions remain for the authors to address:

- The authors describe that the Rab-specificity of PPM1H resides within its flap domain, since a chimaera consisting of the catalytic domain of PPM1J and the flap domain of PPM1H is fully active toward Rab8a. This is also in agreement with the PPM1H-Rab docking model, showing that the flap domain folds over the switch regions and GDP/GTP-binding pocket of Rab. However, intriguingly, the authors show that the flap region also determines specificity for a phosphorylated peptide spanning the region around T72. From the docking model is it less clear how this can be explained. Can the authors attempt to make a docking model of PPM1H with this peptide? This would be very useful to include in the paper. Of course an experimental crystal structure of the PPM1H-peptide complex would even be better, but this reviewer fully understands that the latter most probably has been already attempted without success and will therefore probably not be possible within a reasonable timeframe.

As discussed above, we have provided docking models of both peptide and protein substrates with PPM1H. The newly acquired crosslinking data (Fig 2A-C) are consistent with the overall mode of phospho-Rab8a interactions with PPM1H from 5/6 top hits in the docking calculations.

As a sidenote, it is quite striking that the nucleotide state of Rab8A has a relatively small impact on the PPM1H catalytic parameters (Fig. 2D), considering the position of the flap regions in comparison to the Rab switch regions.

We agree - it is interesting that PPM1H can hydrolyze both. The switch 2 conformation is sensitive to GDP/GTP, although to varying extents depending on the particular Rab protein. There is expected to be greater conformational flexibility in the GDP state. The switch 1 region is also sensitive to GDP/GTP, but in our docked model, it is facing away from the active site and (if correct) may not contribute to PPM1H interactions. In cells, subcellular co-localization of PPM1H to phospho-Rab8a on membranes would presumably involve Rab8a(GTP), but this remains to be demonstrated experimentally. Further insight also requires the structure of a PPM1H-substrate complex.

- For a comparison of the initial rates of WT and mutant PPM1H toward Rab8a and peptide substrates, 16 µM Rab and 32 µM peptide is used. While for the latter this concentration is very close to the KM value, this is unclear for the peptide as kcat and KM values for the peptide are not reported. For a straightforward comparison of the effect of

mutants (e.g. R338A) on Rab and peptide dephosphorylation, substrate concentrations should be used that relate in a similar way to their KM values, e.g. either below or above KM to assess the effect on kcat/KM or kcat, respectively.

We performed assays with peptide concentrations up to 256 μM by overcoming substantial problems of peptide stability (Fig 2E). Similarly, for pRab8a protein substrates (GDP and GTPγS), we collected additional data at 32 μM concentration (Fig 2D). Beyond this amount, the assay is not reliable due to protein precipitation. Regarding kinetics data for pRab10 substrate, this is not currently feasible due to the inability to generate enough phosphorylated protein (see above Referee #1, point 2). However, we have performed a qualitative assay at similar concentrations of pRab8A and pRab10 in a side-by-side comparison (Fig 1D), that demonstrates that PPM1H but not PPM1J efficiently dephosphorylates both substrates.

Minor comments:
- The rationale for making the D288A mutant to increase the crystallisability of the protein is unclear to me (of course, as long as it works it is fine).

Variant 33 to the C-terminus of PPM1H with all loops intact was fortuitously easier to crystallize (uncomplexed) when we introduced the D288A mutation. Our goal at the time was to exploit the variant for crystallization trials of substrate-trapped complexes.

- Figure 2D right panel: the individual datapoints (initial rates at different substrate concentrations) should also be shown, rather than only showing the fitted curve.

Typo's:
- page 7, line 9: "phsopho" should be "phospho"
- page 21 (figure 2 legend): "WT and PPM1H (25 nM) were incubated" should probably be "WT and mutant PPM1H (25 nM) were incubated"
- p22, line 8: "Kcat" should be "kcat".

These above typos have been corrected.

1st Revision - Editorial Decision 10th Aug 2021

Dear Dr. Khan

Thank you for the submission of your revised manuscript to EMBO reports. We have now received the full set of referee reports that is copied below.

As you will see, all referees are very positive about the study and request only minor changes to clarify text and figures.

From the editorial side, there are also a few things that we need before we can proceed with the official acceptance of your study.

1) You have submitted and formatted your manuscript as a Report. Please note that Reports cannot have more than 5 main figures. Your figure panels are already quite data-rich and I do not see much room for merging individual figures to reduce their number to 5. I therefore suggest resubmitting your manuscript in the Article format, i.e., with a separate Results and Discussion section.

2) Figure panels 2D, 2E, 4E and EV3C contain error bars based on n=2. Please use scatter blots showing the individual datapoints instead of the mean and error bars in these cases.

3) Please update the references to the alphabetical Harvard style. The abbreviation 'et al' should be used if more than 10 authors. You can download the respective EndNote file from our Guide to Authors
https://endnote.com/style_download/embo-reports/

4) A callout to Fig. EV1C callout is missing.

5) Figure EV3 is missing. I suggest to relabel Figure EV4 to EV3 and to update all callouts.

6) Data availability section: Please add links that resolve to the respective datasets on PDB and PRIDE in this section. For Zenodo you already have a link, which is fine.

7) Please note our Data citation format if you refer specifically to the reuse of a dataset, as e.g. the structure of pRab8a in the Methods (page 18, ref 38). In this case you could insert a data citation for the structure and in addition cite the paper reporting the structure (see also https://www.embopress.org/page/journal/14693178/authorguide#referencesformat)

8) Please provide the tables listing antibodies and plasmids in one table called "Reagents and Tools Table". This table can be typeset within the methods. A Word or Excel template for this table can be downloaded from our Guide to Authors/Structured methods section.

9) Please note that the Author checklist will be published together with the review process file. Therefore, please remove the information on reviewer passwords from section F-19.

10) During our routine image analysis, which we perform on all revised manuscripts, we noticed that the blots for HA-Rab8A and Phospho-Rab8A Thr72 shown in Fig. 4E look very similar. Checking the source data on Zenodo it seems that the same blot was reprobed but this is not indicated on Zenodo (for Figure 4E 800.tif) in contrast to the description you have for the blots shown in Figure

4F. You might want to add this information, if possible.

For Figure 5F we noticed that the blots for HA (PPMs), LRRK2 total, and GAPDH look identical for the panels showing Rab8 and Rab10. Again, I looked at the source data you deposited on Zenodo and indeed, the blots for HA, LRRK2 and GAPDH do not match the source data for Rab10. It appears that you instead show the blots from the Rab8 data again. Please double-check these panels and the respective source data.

11) Please include all information on funding in the Acknowledgement section.

12) We noticed that you have a paragraph called "Intellectual property rights notice". Could you please clarify this note? If it is required from your funder to include this note in the manuscript, please include it also in the Acknowledgment section. Generally, we encourage Open Access with a CC-BY license at EMBO reports, which would appear to address your funder mandate and obviate the need to publish a separate AAV version by Green OA, which will at best divert online traffic and at worst irritate the reader with two different versions published.

13) Finally, EMBO reports papers are accompanied online by A) a short (1-2 sentences) summary of the findings and their significance, B) 2-3 bullet points highlighting key results and C) a synopsis image that is 550x200-600 pixels large (width x height) in .png format. You can either show a model or key data in the synopsis image. Please note that the size is rather small and that text needs to be readable at the final size. Please send us this information along with the revised manuscript.

We look forward to seeing a final version of your manuscript as soon as possible.

Kind regards,

Martina Rembold, PhD
Senior Editor
EMBO reports

****

Referee #1:

The authors added several new data including crosslinking and docking analysis to show the specific interaction of PPM1H with pRab8a. Although the authors did not present a crystal structure of the complex, they have satisfactorily addressed referee's comments.
Now I think that the revised version is acceptable for publication in EMBO Reports after minor revision below.

Minor points
Page 3 Line 11 from the bottom
"Mutation of the equivalent residue in human PPM1A to alanine (D146A) enabled trapping of a complex of PPM1A with a cyclic phospho-peptide and subsequent structure determination[9]."

In reference # 9, the crystal structure of the PPM1A-cyclic phosphorylated peptide complex was obtained using the "D146E" mutant instead of D146A. The author of reference # 9 stated that

D146A was unable to give crystals of the complex. Therefore, the description should be clarified in this point.

The authors should cite the following two recent comprehensive reviews about PPM family.
https://doi.org/10.1016/j.pharmthera.2020.107622
https://doi.org/10.1016/j.cellsig.2021.110061

Referee #2:

The authors adequately addressed all my points raised in the previous review. The manuscript is technically sound. The authors provide additional data, which clearly strengthen the manuscript. For this reason, I fully support its publication in EMBO Reports.

Minor:
Typo in table 1: R-meas for D288A: value for highest resolution shell contains two decimal separators. Commas missing in the field providing the unit cell dimension for D288A.

Referee #3:

In the revised version of their manuscript the authors have included the results of a number of additional experiments to strengthen their conclusions, including docking experiments, cross-linking experiments to support the docking poses and kinetic experiments. Additionally, they show that PPM1H is a dimer under physiological circumstances, while this dimeric arrangement is not required for catalysis per se. With these additional experiments, and the clarifications given in the revised manuscript and the rebuttal letter, they have sufficiently addressed my remarks and I therefore recommend publication.

Some additional typo's were inserted in the figure legends of the revised document:
- Legend of Figure 1 (A): delete "that connects" in "The loop deletion (188-226) that connects ..."
- Legend title of Figure 2: replace "... analysis suggest flap domain" with "... analysis suggest that the flap domain"
- Legend of Figure 2 (A): delete "page" in "SDS-PAGE page"

Response to reviewers

Referee #1:

The authors added several new data including crosslinking and docking analysis to show the specific interaction of PPM1H with pRab8a. Although the authors did not present a crystal structure of the complex, they have satisfactorily addressed referee's comments.
Now I think that the revised version is acceptable for publication in EMBO Reports after minor revision below.

Minor points
Page 3 Line 11 from the bottom
"Mutation of the equivalent residue in human PPM1A to alanine (D146A) enabled trapping of a complex of PPM1A with a cyclic phospho-peptide and subsequent structure determination[9]."

In reference # 9, the crystal structure of the PPM1A-cyclic phosphorylated peptide complex was obtained using the "D146E" mutant instead of D146A. The author of reference # 9 stated that D146A was unable to give crystals of the complex. Therefore, the description should be clarified in this point.

We have clarified these PPM1A mutants both in the Introduction, and in the Results section (first paragraph).

The authors should cite the following two recent comprehensive reviews about PPM family.

https://doi.org/10.1016/j.pharmthera.2020.107622
https://doi.org/10.1016/j.cellsig.2021.110061

These are now cited in the Introduction.

Referee #2:
The authors adequately addressed all my points raised in the previous review. The manuscript is technically sound. The authors provide additional data, which clearly strengthen the manuscript. For this reason, I fully support its publication in EMBO Reports.

Minor:
Typo in table 1: R-meas for D288A: value for highest resolution shell contains two decimal separators. Commas missing in the field providing the unit cell dimension for D288A.

Corrected.

Referee #3:

In the revised version of their manuscript the authors have included the results of a number of additional experiments to strengthen their conclusions, including docking experiments, cross-

linking experiments to support the docking poses and kinetic experiments. Additionally, they show that PPM1H is a dimer under physiological circumstances, while this dimeric arrangement is not required for catalysis per se. With these additional experiments, and the clarifications given in the revised manuscript and the rebuttal letter, they have sufficiently addressed my remarks and I therefore recommend publication.

Some additional typo's were inserted in the figure legends of the revised document:
- Legend of Figure 1 (A): delete "that connects" in "The loop deletion (188-226) that connects ..."
- Legend title of Figure 2: replace "... analysis suggest flap domain" with "... analysis suggest that the flap domain"
- Legend of Figure 2 (A): delete "page" in "SDS-PAGE page"

Corrected.

2nd Revision - Editorial Decision 27th Aug 2021

Dr. Amir Khan
Trinity College Dublin
School of Biochemistry and Immunology
Trinity Biomedical Sciences Institute
Dublin 2
Ireland

Dear Dr. Khan,

Thank you for incorporating some final changes. I am now very pleased to accept your manuscript for publication in the next available issue of EMBO reports. Thank you for your contribution to our journal.

At the end of this email I include important information about how to proceed. Please ensure that you take the time to read the information and complete and return the necessary forms to allow us to publish your manuscript as quickly as possible.

As part of the EMBO publication's Transparent Editorial Process, EMBO reports publishes online a Review Process File to accompany accepted manuscripts. As you are aware, this File will be published in conjunction with your paper and will include the referee reports, your point-by-point response and all pertinent correspondence relating to the manuscript.

If you do NOT want this File to be published, please inform the editorial office within 2 days, if you have not done so already, otherwise the File will be published by default [contact: emboreports@embo.org]. If you do opt out, the Review Process File link will point to the following statement: "No Review Process File is available with this article, as the authors have chosen not to make the review process public in this case."

Should you be planning a Press Release on your article, please get in contact with emboreports@wiley.com as early as possible, in order to coordinate publication and release dates.

Thank you again for your contribution to EMBO reports and congratulations on a successful publication. Please consider us again in the future for your most exciting work.

Yours sincerely,

Martina Rembold, PhD
Senior Editor
EMBO reports

****

THINGS TO DO NOW:

You will receive proofs by e-mail approximately 2-3 weeks after all relevant files have been sent to

our Production Office; you should return your corrections within 2 days of receiving the proofs.

Please inform us if there is likely to be any difficulty in reaching you at the above address at that time. Failure to meet our deadlines may result in a delay of publication, or publication without your corrections.

All further communications concerning your paper should quote reference number EMBOR-2021-52675V3 and be addressed to emboreports@wiley.com.

Should you be planning a Press Release on your article, please get in contact with emboreports@wiley.com as early as possible, in order to coordinate publication and release dates.

EMBO PRESS

YOU MUST COMPLETE ALL CELLS WITH A PINK BACKGROUND ↓

PLEASE NOTE THAT THIS CHECKLIST WILL BE PUBLISHED ALONGSIDE YOUR PAPER

Corresponding Author Name: Amir R. Khan
Journal Submitted to: EMBO Reports
Manuscript Number: EMBOR-2021-52675V1

USEFUL LINKS FOR COMPLETING THIS FORM

http://www.antibodypedia.com
http://1degreebio.org
http://www.equator-network.org/reporting-guidelines/improving-bioscience-research-repor
http://grants.nih.gov/grants/olaw/olaw.htm
http://www.mrc.ac.uk/Ourresearch/Ethicsresearchguidance/Useofanimals/index.htm
http://ClinicalTrials.gov
http://www.consort-statement.org
http://www.consort-statement.org/checklists/view/32-consort/66-title
http://www.equator-network.org/reporting-guidelines/reporting-recommendations-for-tumo
http://datadryad.org
http://figshare.com
http://www.ncbi.nlm.nih.gov/gap
http://www.ebi.ac.uk/ega
http://biomodels.net/
http://biomodels.net/miriam/
http://jjj.biochem.sun.ac.za
http://oba.od.nih.gov/biosecurity/biosecurity_documents.html
http://www.selectagents.gov/

Reporting Checklist For Life Sciences Articles (Rev. June 2017)

This checklist is used to ensure good reporting standards and to improve the reproducibility of published results. These guidelines are consistent with the Principles and Guidelines for Reporting Preclinical Research issued by the NIH in 2014. Please follow the journal's authorship guidelines in preparing your manuscript.

A- Figures
1. Data
The data shown in figures should satisfy the following conditions:

- the data were obtained and processed according to the field's best practice and are presented to reflect the results of the experiments in an accurate and unbiased manner.
- figure panels include only data points, measurements or observations that can be compared to each other in a scientifically meaningful way.
- graphs include clearly labeled error bars for independent experiments and sample sizes. Unless justified, error bars should not be shown for technical replicates.
- if n< 5, the individual data points from each experiment should be plotted and any statistical test employed should be justified
- Source Data should be included to report the data underlying graphs. Please follow the guidelines set out in the author ship guidelines on Data Presentation.

2. Captions

Each figure caption should contain the following information, for each panel where they are relevant:

- a specification of the experimental system investigated (eg cell line, species name).
- the assay(s) and method(s) used to carry out the reported observations and measurements
- an explicit mention of the biological and chemical entity(ies) that are being measured.
- an explicit mention of the biological and chemical entity(ies) that are altered/varied/perturbed in a controlled manner.

- the exact sample size (n) for each experimental group/condition, given as a number, not a range;
- a description of the sample collection allowing the reader to understand whether the samples represent technical or biological replicates (including how many animals, litters, cultures, etc.).
- a statement of how many times the experiment shown was independently replicated in the laboratory.
- definitions of statistical methods and measures:
  - common tests, such as t-test (please specify whether paired vs. unpaired), simple χ^2 tests, Wilcoxon and Mann-Whitney tests, can be unambiguously identified by name only, but more complex techniques should be described in the methods section;
  - are tests one-sided or two-sided?
  - are there adjustments for multiple comparisons?
  - exact statistical test results, e.g., P values = x but not P values < x;
  - definition of 'center values' as median or average;
  - definition of error bars as s.d. or s.e.m.

Any descriptions too long for the figure legend should be included in the methods section and/or with the source data.

In the pink boxes below, please ensure that the answers to the following questions are reported in the manuscript itself. Every question should be answered. If the question is not relevant to your research, please write NA (non applicable). We encourage you to include a specific subsection in the methods section for statistics, reagents, animal models and human subjects.

B- Statistics and general methods

Please fill out these boxes ↓ (Do not worry if you cannot see all your text once you press return)

1.a. How was the sample size chosen to ensure adequate power to detect a pre-specified effect size?	For the immunoblot cell based assays each experiment was repeated at least three times, additionally with duplicates or triplicates for each condition within an experiment, with the presented blot used for quantitation. The in vitro dephosphorylation assays were performed at least in triplicates, with the mean +/-SE (as error bars) from all experiments presented.
1.b. For animal studies, include a statement about sample size estimate even if no statistical methods were used.	NA
2. Describe inclusion/exclusion criteria if samples or animals were excluded from the analysis. Were the criteria pre-established?	NA
3. Were any steps taken to minimize the effects of subjective bias when allocating animals/samples to treatment (e.g. randomization procedure)? If yes, please describe.	NA
For animal studies, include a statement about randomization even if no randomization was used.	NA
4.a. Were any steps taken to minimize the effects of subjective bias during group allocation or/and when assessing results (e.g. blinding of the investigator)? If yes please describe.	NA
4.b. For animal studies, include a statement about blinding even if no blinding was done	NA
5. For every figure, are statistical tests justified as appropriate?	NA
Do the data meet the assumptions of the tests (e.g., normal distribution)? Describe any methods used to assess it.	NA
Is there an estimate of variation within each group of data?	NA

Is the variance similar between the groups that are being statistically compared?	NA

C- Reagents

6. To show that antibodies were profiled for use in the system under study (assay and species), provide a citation, catalog number and/or clone number, supplementary information or reference to an antibody validation profile. e.g., Antibodypedia (see link list at top right), 1DegreeBio (see link list at top right).	Development and validation of the Rab8A pT72 (Abcam, ab230260) and Rab10 pT73 (Abcam, ab243293) anibodies was described in PMID 29127256. LRRK2 antibody (Neuromab, 73-253) validation profile is available on Antibodypedia.
7. Identify the source of cell lines and report if they were recently authenticated (e.g., by STR profiling) and tested for mycoplasma contamination.	HEK293 cell line was obtained from ATCC (CRL-1573) and regularly tested for mycoplasma contamination.

* for all hyperlinks, please see the table at the top right of the document

D- Animal Models

8. Report species, strain, gender, age of animals and genetic modification status where applicable. Please detail housing and husbandry conditions and the source of animals.	NA
9. For experiments involving live vertebrates, include a statement of compliance with ethical regulations and identify the committee(s) approving the experiments.	NA
10. We recommend consulting the ARRIVE guidelines (see link list at top right) (PLoS Biol. 8(6), e1000412, 2010) to ensure that other relevant aspects of animal studies are adequately reported. See author guidelines, under 'Reporting Guidelines'. See also: NIH (see link list at top right) and MRC (see link list at top right) recommendations. Please confirm compliance.	NA

E- Human Subjects

11. Identify the committee(s) approving the study protocol.	NA
12. Include a statement confirming that informed consent was obtained from all subjects and that the experiments conformed to the principles set out in the WMA Declaration of Helsinki and the Department of Health and Human Services Belmont Report.	NA
13. For publication of patient photos, include a statement confirming that consent to publish was obtained.	NA
14. Report any restrictions on the availability (and/or on the use) of human data or samples.	NA
15. Report the clinical trial registration number (at ClinicalTrials.gov or equivalent), where applicable.	NA
16. For phase II and III randomized controlled trials, please refer to the CONSORT flow diagram (see link list at top right) and submit the CONSORT checklist (see link list at top right) with your submission. See author guidelines, under 'Reporting Guidelines'. Please confirm you have submitted this list.	NA
17. For tumor marker prognostic studies, we recommend that you follow the REMARK reporting guidelines (see link list at top right). See author guidelines, under 'Reporting Guidelines'. Please confirm you have followed these guidelines.	NA

F- Data Accessibility

18: Provide a "Data Availability" section at the end of the Materials & Methods, listing the accession codes for data generated in this study and deposited in a public database (e.g. RNA-Seq data: Gene Expression Omnibus GSE39462, Proteomics data: PRIDE PXD000208 etc.) Please refer to our author guidelines for 'Data Deposition'.<br><br>Data deposition in a public repository is mandatory for:<br>a. Protein, DNA and RNA sequences<br>b. Macromolecular structures<br>c. Crystallographic data for small molecules<br>d. Functional genomics data<br>e. Proteomics and molecular interactions	All Plasmids, antibodies and proteins (including datasheets and sequence information) that we have generated for this study can be requested and information downloaded from MRC PPU Reagents and Services (https://mrcppureagents.dundee.ac.uk/). The structures of PPM1H phosphatase have been deposited in the Protein Data Bank with codes 7kpr, 7l4j, 7l4i, and 7n0z. Six detailed step-by-step protocols were made available through protocols.io, describing expression and purification of PPM1H, expression and purification of phosphorylated Rab8a and Rab10, the Malachite green phosphatase activity assay, LRRK2 and phospho-Rab immunoblotting assay, cross-linking/MS analysis PPM1H and phopsho-Rab8A, with a doi numbers listed in each method description.
19. Deposition is strongly recommended for any datasets that are central and integral to the study; please consider the journal's data policy. If no structured public repository exists for a given data type, we encourage the provision of datasets in the manuscript as a Supplementary Document (see author guidelines under 'Expanded View' or in unstructured repositories such as Dryad (see link list at top right) or Figshare (see link list at top right).	All primary data associated with each figure has been deposited in the Zenodo data repository with a doi number 10.5281/zenodo.5045023. The mass spectrometry proteomics data have been deposited to the ProteomeXchange Consortium via the PRIDE partner repository with the dataset identifier PXD026367.
20. Access to human clinical and genomic datasets should be provided with as few restrictions as possible while respecting ethical obligations to the patients and relevant medical and legal issues. If practically possible and compatible with the individual consent agreement used in the study, such data should be deposited in one of the major public access-controlled repositories such as dbGAP (see link list at top right) or EGA (see link list at top right).	NA
21. Computational models that are central and integral to a study should be shared without restrictions and provided in a machine-readable form. The relevant accession numbers or links should be provided. When possible, standardized format (SBML, CellML) should be used instead of scripts (e.g. MATLAB). Authors are strongly encouraged to follow the MIRIAM guidelines (see link list at top right) and deposit their model in a public database such as Biomodels (see link list at top right) or JWS Online (see link list at top right). If computer source code is provided with the paper, it should be deposited in a public repository or included in supplementary information.	Docking models of the enzyme and substrate complex were generated using the default parameters in the Haddock web site. Details of the input files and restraints are provided in the Methods section. Coordinates for the docked complex have been deposited in the Biomodels database with accession code MODEL2108130001.

G- Dual use research of concern

22. Could your study fall under dual use research restrictions? Please check biosecurity documents (see link list at top right) and list of select agents and toxins (APHIS/CDC) (see link list at top right). According to our biosecurity guidelines, provide a statement only if it could.	NA
